# Supplementary material for: Identification of a specific set of genes predicting obesity before phenotype appearance
Source: iScience. 2025 Apr 8;28(5):112377. doi: 10.1016/j.isci.2025.112377 (PMC12053654; doi:10.1016/j.isci.2025.112377)
Supplement: Document S1. Figures S1–S4 [file mmc1.pdf]

iScience, Volume *28*

Supplemental information

Identification of a specific set of genes predicting obesity before phenotype appearance

Céline Jousse, Laurent Parry, Gwendal Cueff, Marion Brandolini-Bunlon, Jérémy Tournayre, Alain Bruhat, Anne-Catherine Maurin, Cyrielle Vituret, Julien Averous, Yuki Muranishi, and Pierre Fafournoux

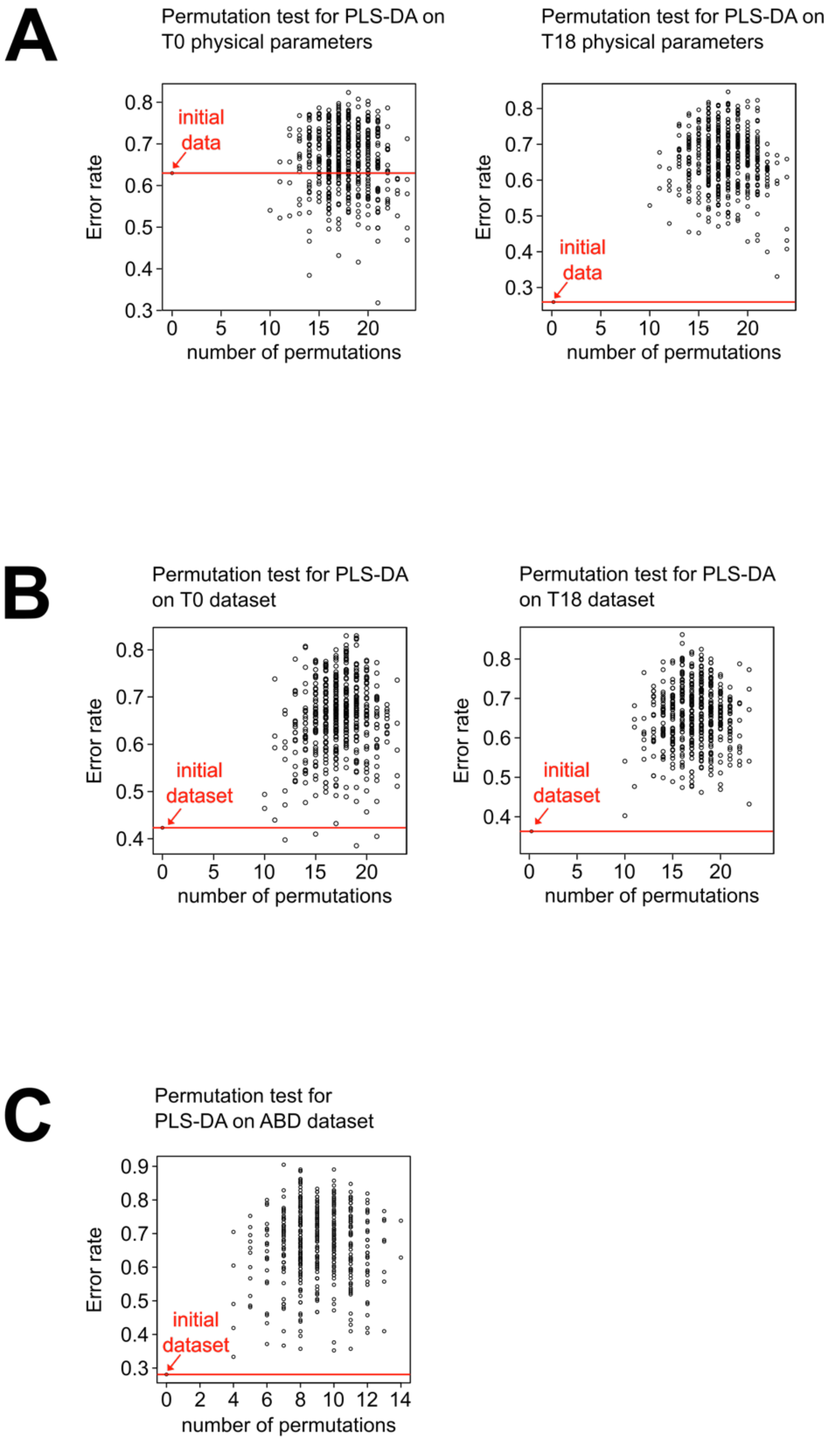
A
Permutation test for PLS-DA on
T0 physical parameters
Error rate
0.8
0.7
0.6
0.5
0.4
0.3
initial
data
0
5
10
15
20
number of permutations
Permutation test for PLS-DA on
T18 physical parameters
Error rate
0.8
0.7
0.6
0.5
0.4
0.3
initial
data
0
5
10
15
20
number of permutations
B
Permutation test for PLS-DA
on T0 dataset
Error rate
0.8
0.7
0.6
0.5
0.4
initial
dataset
0
5
10
15
20
number of permutations
Permutation test for PLS-DA
on T18 dataset
Error rate
0.8
0.7
0.6
0.5
0.4
initial
dataset
0
5
10
15
20
number of permutations
C
Permutation test for
PLS-DA on ABD dataset
Error rate
0.9
0.8
0.7
0.6
0.5
0.4
0.3
initial
dataset
0
2
4
6
8
10
12
14
number of permutations

Supplementary Figure S1:

The efficiency of the various models obtained was tested using a permutation test. A permutation test is used to assess whether the observed discrimination between groups is statistically significant or could have occurred by chance. The permutation test for PLS-DA involved randomly permuting the group membership of the observations and recalculating the PLS-DA model for each permutation. This procedure was repeated 500 time. The performance of each of the permuted models obtained was assessed by their classification error rate and compared to the error rate of the true model (indicated in red).

A: Permutation test for PLS-DA with physical parameters measured at T0 and T18. At T0, the error rates obtained with permuted models were generally as “good” as those obtained with the real dataset (left panel), indicating that it is not possible to create a discriminant model with the T0 physical data. On the other hand, PLS-DA and permutation tests performed on the T18 physical data (i.e. after the HFD-challenge, right panel) confirmed that the three groups identified are indeed different and discrimination can be made based on physical parameters.

B: Permutation test for PLS-DA with T0 and T18 gene expression datasets

At both T0 and T18, the error rates obtained with permuted models were higher than the original model showing that the PLS-DA models were valid.

C: Permutation test for PLS-DA on ABD dataset.

All error rate values of permuted models were higher than the original model showing that the PLS-DA model was valid.

	A	B	D
Glucose (mmol/L)			
Number of values	26	32	24
Mean	8.417	7.599	8.564
Std. Error of Mean	0.2441	0.1286	0.2548
p-value A vs B / A vs D		0.013	ns
Cholesterol (mmol/L)			
Number of values	15	20	23
Mean	2.293	2.179	2.204
Std. Error of Mean	0.08365	0.08096	0.08265
p-value A vs B / A vs D		ns	ns
Triglycerides (mmol/L)			
Number of values	14	18	20
Mean	0.65	0.4917	0.809
Std. Error of Mean	0.02722	0.01984	0.04611
p-value A vs B / A vs D		0.011	<0.0001
HDL (mmol/L)			
Number of values	14	20	24
Mean	2.433	2.48	2.446
Std. Error of Mean	0.04602	0.0683	0.0835
p-value A vs B / A vs D		ns	ns

Supplementary Figure S2:
Plasma metabolites parameters of 5-mo-old male mice from group A, B, and D, were measured in plasma of over-night starved animals.

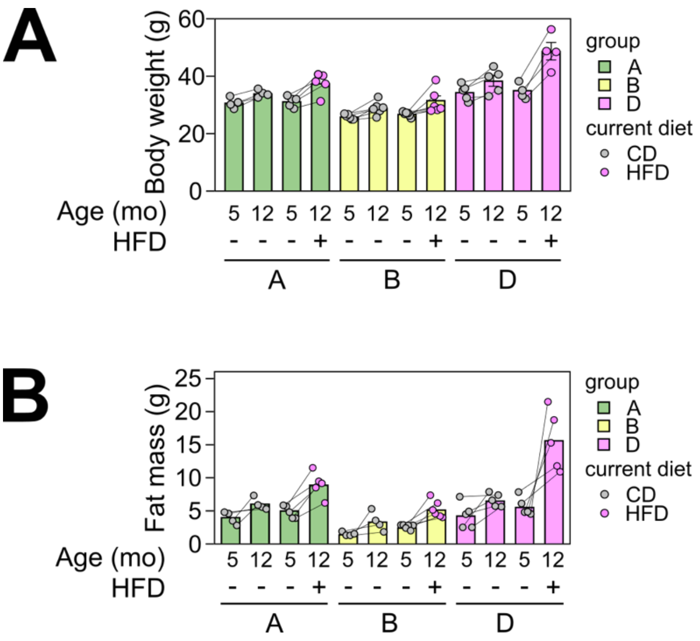

<u>Supplementary Figure S3:</u>

Body weight and fat mass change following a 7-months HFD-challenge on groups A, B and D: 5-months-old male offspring from groups A, B and D were fed either a CD or a HFD from the age of 5 to 12-month (7 months long HFD-challenge) as described Figure 3C. Body weight and fat mass were recorded at the beginning and at the end of the HFD-challenge. **A: Body weight. B: Fat mass.** n = 4 to 6

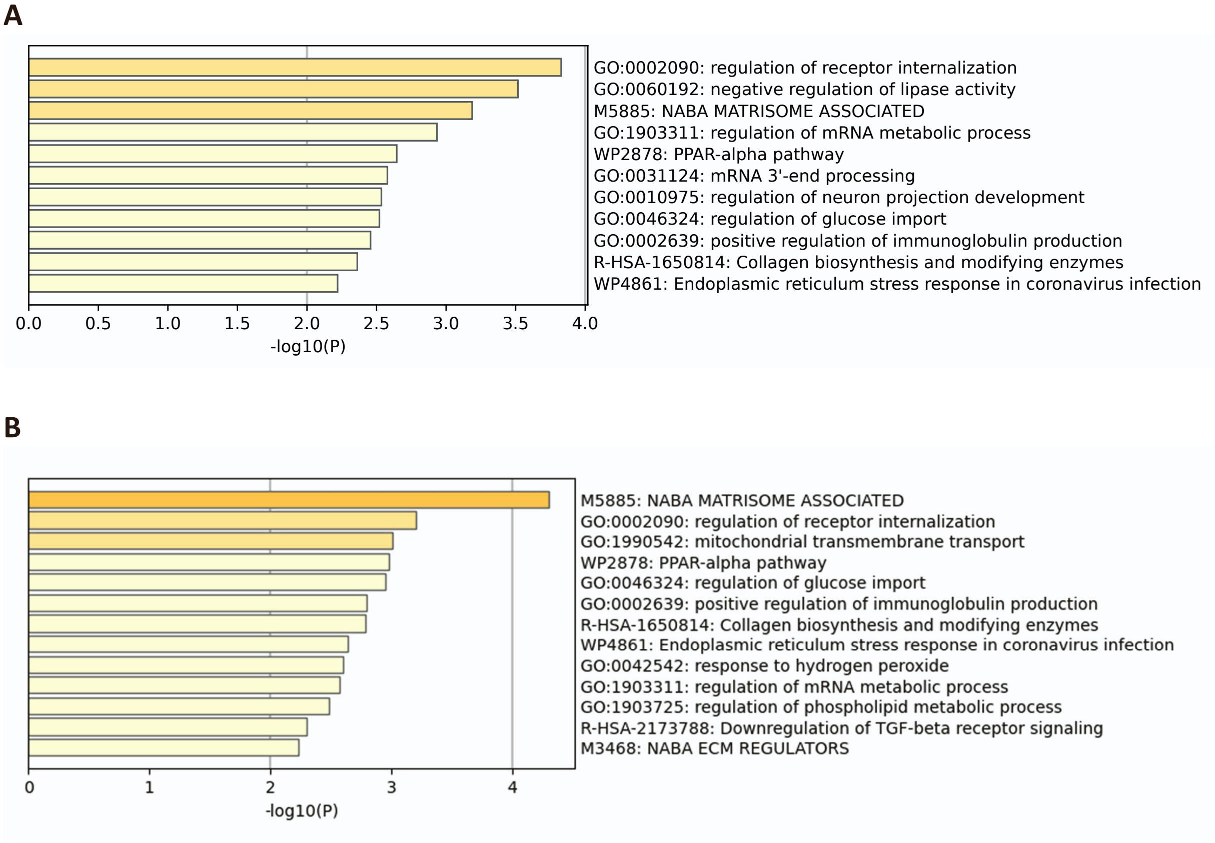

Supplementary Figure S4: Gene Ontology (GO) enrichment analysis

The GO enriched terms are colored by p-value [9]. A: enrichment analysis performed on the list of 143 genes (see Table S1, column B). B: enrichment analysis performed on the list of 109 genes (see Table S1, column D)

Supplementary References

[1] Y.-H. Lee, « Transcription profiling of human abdominal subcutaneous adipocytes from obese and non-obese Pima Indians ». 2007. ArrayExpress: E-GEOD-2508. https://www.ebi.ac.uk/biostudies/arrayexpress/studies/E-GEOD-2508

[2] Y. H. Lee *et al.*, « Microarray profiling of isolated abdominal subcutaneous adipocytes from obese vs non-obese Pima Indians: increased expression of inflammation-related genes », 2005. Diabetologia *48*, 1776–1783. doi: 10.1007/s00125-005-1867-3.

[3] C. M. Aguilera, « Transcription profiling of human omental adipose tissue from obese children ». 2008. ArrayExpress: E-GEOD-9624. https://www.ebi.ac.uk/biostudies/arrayexpress/studies/E-GEOD-9624

[4] C. Aguilera, C. Gomez-Llorente, I. Tofe, M. Gil-Campos, R. Cañete, et Á. Gil, « Genome-Wide Expression in Visceral Adipose Tissue from Obese Prepubertal Children », *IJMS*, vol. 16, n° 12, Art. n° 12, avr. 2015, doi: 10.3390/ijms16047723.

[5] S. Dankel, G. Mellgren, et J.-I. Bjune, « Microarray of isolated adipocytes from subcutaneous adipose tissue of severely obese patients compared to lean controls ». 2018. ArrayExpress: E-MTAB-6728. https://www.ebi.ac.uk/biostudies/arrayexpress/studies/E-MTAB-6728

[6] J.-I. Bjune *et al.*, « IRX5 regulates adipocyte amyloid precursor protein and mitochondrial respiration in obesity », *Int J Obes*, vol. 43, n° 11, Art. n° 11, nov. 2019, doi: 10.1038/s41366-018-0275-y.

[7] F. Rey, « Transcriptional deregulation in subcutaneous adipose tissue from severely obese patients is associated with cancer: focus on gender differences and role of type 2 diabetes (human) ». 2021. GEO: GSE166047. https://www.ncbi.nlm.nih.gov/bioproject/699024

[8] F. Rey *et al.*, « Transcriptome Analysis of Subcutaneous Adipose Tissue from Severely Obese Patients Highlights Deregulation Profiles in Coding and Non-Coding Oncogenes », *IJMS*, vol. 22, n° 4, Art. n° 4, févr. 2021, doi: 10.3390/ijms22041989.

[9] Y. Zhou *et al.*, « Metascape provides a biologist-oriented resource for the analysis of systems-level datasets », *Nat Commun*, vol. 10, n° 1, Art. n° 1, déc. 2019, doi: 10.1038/s41467-019-09234-6.
